# State Medical Marijuana Laws and Initiation of Cigarettes among Adolescents in the U.S., 1991–2015

**DOI:** 10.26828/cannabis/2021.01.004

**Published:** 2021-04-22

**Authors:** Abhery Das, Julie K. Johnson, Gregory A. Hard, Abenaa A. Jones

**Affiliations:** 1 Program in Public Health, University of California, Irvine, 653 East Peltason Drive, Irvine, CA 92617; 2 Cannabis Policy Research Center of Excellence, Research Department, Cannabis Control Commission, Commonwealth of Massachusetts, 2 Washington Square, 2nd Floor, Worcester, MA 01604; 3 MGH Institute of Health Professions, 36 First Avenue, Boston MA 02129; 4 Department of Human Development and Family Studies, The Pennsylvania State University, 105 Health and Human Development Building, University Park, PA 16802, USA

**Keywords:** Medical Marijuana Laws, cigarette initiation, adolescents, drug policy, marijuana

## Abstract

**Objective.:**

Although cigarette use has declined among adolescents, marijuana use has increased in subgroups of this population. The association between medical marijuana laws (MMLs) and cigarette initiation among adolescents, however, needs further examination. We investigated the association between MMLs and age of cigarette initiation and stratified findings by gender, race/ethnicity, and state dispensary status.

**Method.:**

Data were from N=939,725 adolescents in 9^th^-12^th^ grade living in 46 states who participated in the Youth Risk Behavior Surveillance System between 1991–2015. Participants were asked the age they first smoked a cigarette and other sociodemographic characteristics. States were categorized as MML states if they had legalized marijuana for medicinal purposes by 2015. We used a difference-in-difference methodology and logistic regressions to assess the relationship between MMLs and cigarette initiation.

**Results.:**

Our results indicate lower odds of initiating cigarettes, in every age group (8 years old or younger, 9–10, 11–12, 13–14, 15–16, 17 years old or older) in states with MMLs when compared to non-MML states. After stratification, we find lower odds of cigarette initiation in certain age groups by gender, race/ethnicity, and state dispensary status. We report no difference in state MML implementation and age of cigarette initiation among Hispanic adolescents in every age group, and Black adolescents 8 years or younger and 17 years or older.

**Conclusions.:**

Cigarette initiation has decreased among adolescents in MML states compared with those in non-MML states. Further research should evaluate how MMLs and recreational marijuana policies are associated with e-cigarette initiation and use.

The landscape of marijuana legalization has changed significantly over the past two decades in the United States (State Medical Marijuana Laws, 2020). Currently, medical marijuana laws (MMLs) have passed in 36 states (State Medical Marijuana Laws, 2020). Policies legalizing marijuana may influence substance use, such as cigarettes, in the broader population. Adolescents remain particularly vulnerable as tobacco use is commonly established during youth (Office on Smoking and Health [OSH], 2020a).

Following the enactment of MMLs, marijuana and other substances—such as cigarettes—may have a complementary relationship wherein both marijuana and substance use increases (Cerdá et al., 2018; Guttmannova et al., 2019). This may occur if co-use produces a synergistic psychoactive effect or if marijuana remains a gateway for other substances (Cerdá et al., 2018). Alternatively, marijuana may substitute other substances if they have similar psychoactive properties or if marijuana becomes more accessible (Cerdá et al., 2018; Guttmannova et al., 2019).

In 2019, 2.3% of adolescents reported using cigarettes in the past 30 days – a decrease from 4.3% in 2011 (OSH, 2020a). Disparities also exist among youth, with some racial/ethnic groups reporting ever use of tobacco products at higher rates than non-Hispanic Whites (Odani, 2018). Studies reported that adolescents more often use marijuana and cigarettes concurrently as opposed to either substance alone (Lanza et al., 2015). A systematic review indicated that 90% of cross-sectional studies find a positive relation between cigarette smoking and marijuana use in adolescents (Ramo et al., 2012). Adolescents who use marijuana were six to 12 times more likely to smoke cigarettes (Ramo et al., 2012). Additionally, adolescents who use tobacco were 52 times more likely to use marijuana (Ramo et al., 2012).

Previous research on MML and cigarette use among adolescents has found conflicting results by age. Cerdá et al. (2018) reported that MMLs were associated with a decrease in cigarette use among 8^th^ graders, but an increase among 12^th^ graders. Wang et al. (2016) reported that statewide legalization of medical marijuana was associated with cigarette and marijuana co-use, with 12–17 year olds having three times the likelihood of nicotine dependence. Wen et al. (2015) found a greater percentage of daily and non-daily smokers between 12–20 year olds in MML states as opposed to non-MML states. The relation between MMLs and cigarette initiation rather than cigarette use among adolescents, however, remains unknown.

Youth cigarette initiation trends have changed over the past two decades. (Cantrell et al., 2018). Some studies have found that the majority of initiation occurs before 18 years old (OSH, 2012). Cantrell et al. (2018) reported that cigarette initiation decreased for those aged 12–14 and 15–17 among all race/ethnicities and genders between 2002 and 2015. Azagba et al. (2020) found that age of cigarette initiation increased, specifically among female and high school students. Given the changing nature of cigarette use in the United States and the potential for a substitution effect between marijuana and tobacco following MMLs, we examined whether MMLs are associated with age of cigarette initiation by race/ethnicity, gender, and state marijuana dispensary status.

## METHODS

### Study Population

We obtained data from the Youth Risk Behavior Surveillance System (YRBSS) in 46 states from 1991 – 2015. This surveillance system monitors health risk behaviors among youth in the United States (Center for Disease Control [CDC], 1995 – 2015). The YRBSS is a cross-sectional survey, of 9^th^ to 12^th^-grade students conducted by the CDC and national, state, and local agencies. Minnesota, Oregon, and Washington did not participate in the survey (CDC, 1995 – 2015). We were unable to obtain data on Hawaii due to the data application process. YRBSS draws state samples from a two-stage cluster sampling design that first randomly selects schools to participate and then randomly selects classes within those schools. YRBSS attains overall response rates of ≥ 60%. We weighted the data to obtain estimates representative of 9–12 grade students within each state. We therefore only included states that allowed for weighting (Brener et al., 2013).

### Measures

For our outcome variable, we used the YRBSS question, “How old were you when you first smoked a whole cigarette for the first time?” (CDC, 1995 – 2015). We created binary variables for the age of cigarette initiation, including 8 years old or younger, 9–10 years old, 11–12 years old, 13–14 years old, 15–16 years old, and 17 years old or older. These age groups may not coincide with the age of the survey participant as participants may have initiated cigarettes at earlier ages. As our exposure variable, we coded states that had enacted MMLs as a binary variable (0 for before MML; 1 for after MML) (State Medical Marijuana Laws, 2020). States that did not enact MMLs were coded as ‘0’ for the entire study period. As control variables, we included the demographic characteristics of the survey participants including age, gender, and race/ethnicity (non-Hispanic White, non-Hispanic Black, Hispanic, and Other) and state-year fixed effects. We also included variables indicating whether states allowed marijuana dispensaries to operate (0 for dispensaries not allowed; 1 for dispensaries allowed) and whether states had active dispensaries (0 for no active dispensaries; 1 for active dispensaries) for each year (State Medical Marijuana Laws, 2020).

### Statistical Analysis

We used pre-post, difference-in-difference (DID) methodologies to examine both change in cigarette initiation in states before and after MML enactment with non-MML states, as well as states that never enacted MMLs with states that enacted MMLs in the study period. Widely used in health policy research and in previous MML literature, DID controls for secular trends that do not change over time. This quasi-experimental research design uses MML states as the ‘treatment group’ and both non-MML states and MML states prior to MML enactment as the ‘comparison group’. We used logistic regression analyses to examine the relations between MMLs and the age of cigarette initiation overall and among race/ethnicity, gender, and state marijuana dispensary status.

The DID methodology assumes parallel trends in the outcome among the treatment and control groups prior to implementation of the ‘treatment’ or policy. This assumption was tested by creating line graphs to visualize outcomes over time and confirm parallel trends. We confirmed that MML and non-MML states had parallel trends in the outcome prior to the implementation of these policies. Our state fixed effects specification accounted for differential characteristics among states. We also specified year fixed effects to control for year-specific factors that may correspond with the outcome of interest. We used Stata 15 MP for all analyses. Additionally, we used Stata’s “svyset” command to account for YRBSS sampling and weighting procedures. We used robust standard errors to adjust for heteroscedasticity in the residuals and any correlation of errors within the specified clusters (i.e., states). Johns Hopkins Bloomberg School of Public Health Institutional Review Board deemed this study exempt because it used publicly available, deidentified data.

## RESULTS

### Sample Characteristics

The study sample comprised 939,725 adolescents. Adolescent race/ethnicity included non-Hispanic White (61.13%), non-Hispanic Black (14.53%), Hispanic (14.13%), and Other race/ethnicity (10.21%) (Table 1). Females and males comprised 50.88% and 49.12% of the study population, respectively. Survey participants were aged 12 years old or younger (0.37%), 13 years old (0.36%), 14 years old (12.19%), 15 years old (26.16%), 16 years old (26.60%), 17 years old (22.71%), and 18 years old or older (11.62%) (Table 1). Table 2 details states that enacted MMLs between 1991 – 2015, year of enactment, and the years of data before and after enactment. Among the 46 states, 19 of them enacted MMLs during the study period.

### Initiation of Cigarette Use

After adjusting by state, year, and individual demographics (age, gender, and race/ethnicity), the analyses indicate a lower odds of initiating cigarettes among all age categories in states with enacted MMLs compared to states without MMLs in the year of data collection (Table 3). In Table 3, we find a lower odds of cigarette initiation among all age strata for females and all age strata for males except for those aged 17 years old or older.

### Initiation of Cigarette Use by Race/Ethnicity

Among the racial/ethnic categories, our results indicate a lower odds of cigarette initiation in MML states among all non-Hispanic White age strata and all non-Hispanic Black age strata, except for 8 years old or younger and 17 years old or older (Table 3). We did not find significant results for any age categories among Hispanic adolescents. Lastly, our results indicate a lower odds of cigarette initiation in MML states among Other race/ethnicity except for 13–14-year-olds (Table 3).

In Table 3, we report lower odds of cigarette initiation in MML states with dispensaries allowed among all age strata, except for those aged 8 years old or younger. We also find lower odds of cigarette initiation in MML states with active dispensaries, among those aged 9–10, 11–12, and 13–14 years old.

## DISCUSSION

Our results indicate a lower odds of initiating cigarettes in MML states among all adolescent age groups. After stratifying by gender, race/ethnicity, and state dispensary status, we also find lower odds of cigarette initiation among certain age strata.

As suggested by other scholars, the ‘substitution effect’ may play a role in the decrease in cigarette initiation after enactment of MMLs. Marijuana may provide the same psychoactive properties as cigarettes or become more accessible to adolescents in states with MMLs (Cerdá et al., 2018). However, previous studies have also found conflicting results as to marijuana use among adolescents following MML implementation (Cerdá et al., 2018; Johnson et al., 2017, 2021; Ladegard et al., 2020). Alternatively, adolescents may also substitute cigarettes with electronic cigarettes (“e-cigarettes” or “vaping”; OSH, 2020b). E-cigarettes are popular among adolescents as more than 19% of high school students report using e-cigarettes in the past 30 days (Wang, 2020). There also may be a ‘gateway effect’ of e-cigarette use leading to use of conventional cigarettes, especially among adolescents (Morgenstern et al., 2018). One study conducted among 10^th^ graders reported that e-cigarette users more commonly experiment with conventional cigarettes (Morgenstern et al., 2018). Further investigation into whether MMLs correspond with e-cigarette use or conventional cigarette initiation, by way of e-cigarettes, is warranted as research on this issue remains scarce. Recent work reported, however, that adolescents have increased marijuana use with vaping devices following cannabis legalization (Borodovsky, 2017; Nicksic et al., 2020).

Additionally, increase in price, due to tobacco taxes, may deter adolescents from smoking cigarettes (Hawkins et al., 2016). One study on whether cigarette price influences adolescent experimentation found no relation (Emery et al., 2001). The study suggested that because adolescent experimenters consume few cigarettes, they are not affected by the price of cigarettes (Emery et al., 2001). Other studies indicated that higher tobacco tax rates were associated with reduced cigarette initiation and days smoked among adolescents (Apollonio et al., 2021; Forster et al., 2007). State-level differences in tobacco tax policies may influence adolescent age groups differently (Forster et al., 2007). As with our findings, certain age strata may respond differently to state-level policies. Further research would benefit from better understanding how tobacco tax laws may influence specific age groups.

Current media campaigns and communications aimed at reducing tobacco initiation among adolescents may also deter cigarette initiation (The Community Guide, 1999). Several studies have found that an increase in mass-health communications – through television, radio, and print – show a reduction in tobacco use (The Community Guide, 1999). Future research may also examine how media campaigns and communications about marijuana policy and e-cigarettes may correspond with adolescent substance use.

Marijuana policy in the U.S. continues to change as more states legalize medical and recreational use of marijuana and decriminalize marijuana possession. Currently, 15 states have legalized recreational marijuana for adults. These policy changes may increase accessibility of cannabis to adolescents, therefore supporting the ‘substitution effect’ of marijuana use in place of conventional cigarettes (Hartman, 2021). Our findings further indicate the potential for substitution – rather than a complementary relationship – between cigarettes and marijuana. The increase in marijuana use among adolescent subgroups following cannabis legalization, however, remains concerning. Previous studies have found that adolescent marijuana use precedes increases in substance use disorders, as well as adverse mental health and cognitive development in adulthood (Ladegard et al., 2020). This warrants further research on the mental health and cognitive spillover effects of the implementation of such policies.

### Limitations

Our study has limitations. We did not include additional policies, such as recreational marijuana legalization or tobacco laws, that may influence cigarette initiation in adolescents. The analysis attempted to address this potential confounding by using state and year fixed effects and a difference-in-difference study design (Angrist & Pischke, 2008). Beginning in 2013, states began prohibiting tobacco product sales to persons less than 21 years old – also referred to as T21 laws – to reduce adolescent tobacco use (Marynak, 2020). Federal policy also raised the minimum legal sales age of tobacco products to 21 in 2019 (Marynak, 2020). Given the potential relation between T21 laws and MMLs or adolescent tobacco use, further research would benefit from understanding this relationship. Another limitation of the study includes the use of a general MML exposure variable with one provision category (allowed and active dispensaries). We did not include provision categories for MMLs such as home cultivation, caregivers, possession, and patient registration as previous research has not found a relation between these provisions and adolescent substance use (Johnson et al., 2017, 2021). Nonetheless, further research should investigate how such provisions may affect adolescent cigarette initiation as MMLs become enacted across the US.

### Strengths

Despite any weaknesses, our study has significant strengths, including the use of the YRBSS, a population-representative sample of students that is used extensively in literature (Brener et al., 2013). Its long history and consistent methodology allow for analysis of long-term trends in adolescent health behaviors (Brener et al., 2013). In this study, we have included 25 years of data from 46 states with a large sample of over 900,000 adolescents. Another strength of this study includes the use of the difference-in-difference methodology. This quasi-experimental design removes bias in the post-intervention period comparisons resulting from permanent differences between the ‘treatment’ and ‘control’ groups (Angrist & Pischke, 2008).

### Conclusion

Cigarette initiation has decreased among adolescents living in states that enacted MMLs compared to those residing in non-MML states. This finding supports the possibility of a ‘substitution effect’ in that adolescents may substitute marijuana for cigarettes. Given the significant increases in vaping among youth, research should examine whether MMLs and recreational marijuana policies are associated with e-cigarette use. Further research should also evaluate the possible mental health and cognitive effects associated with heightened marijuana use among adolescents following cannabis legalization.

## Figures and Tables

**Table 1.** Sample characteristics (race/ethnicity, gender, and age) for participants in the YRBSS from 46 U.S. states, 1991–2015 (N = 939,725).

Characteristics	N (%)
**Race/Ethnicity**	
White^a^	574,454 (61.1%)
Black^a^	136,543 (14.5%)
Hispanic	132,783 (14.1%)
Other^a^	95,945 (10.2%)
**Gender**	
Female	478,132 (50.9%)
Male	461,593 (49.1%)
**Age**	
12 years or younger	3,440 (0.4%)
13 years old	3,426 (0.4%)
14 years old	114,522 (12.2%)
15 years old	245,825 (26.2%)
16 years old	249,933 (26.6%)
17 years old	213,367 (22.7%)
18 years old or older	109,212 (11.6%)

a Non-Hispanic

**Table 2.** States that enacted medical marijuana laws (MMLs), enactment year, and years of pre- and post- enactment data, 1991–2015.

Year MML Enacted	State	Years of Data Pre-MML Enactment	Years of Data Post-MML Enactment
1996	California (CA)	0	1
1998	Alaska (AK)	1	5
1999	Maine (ME)	2	8
2000	Colorado (CO)	0	3
2000	Nevada (NV)	4	7
2004	Montana (MT)	6	6
2004	Vermont (VT)	0	3
2006	Rhode Island (RI)	4	5
2007	New Mexico (NM)	2	5
2008	Michigan (MI)	6	4
2010	Arizona (AZ)	4	3
2010	New Jersey (NJ)	3	2
2011	Delaware (DE)	5	2
2012	Connecticut (CT)	5	2
2012	Massachusetts (MA)	7	2
2013	Illinois (IL)	5	2
2013	New Hampshire (NH)	7	2
2014	New York (NY)	8	1
2014	Maryland (MD)	5	1

**Table 3.** Logistic regression results predicting odds of cigarette initiation among adolescents, by age strata, in states with enacted MMLs compared to states without MMLs in the year of data collection, 1991–2015.^a^

Age of Cigarette Initiation	Adjusted OR	(95%CI)
8 years old or younger	0.86***	(0.79– 0.94)
9–10 years old	0.72***	(0.65– 0.78)
11–12 years old	0.77***	(0.71– 0.83)
13–14 years old	0.79***	(0.74– 0.84)
15–16 years old	0.85***	(0.80– 0.90)
17 years old or older	0.87*	(0.77– 0.99)
**Gender**		
**Female**		
8 years old or younger	0.83**	(0.73– 0.95)
9–10 years old	0.69	(0.60– 1.79)
11–12 years old	0.74***	(0.67– 0.83)
13–14 years old	0.79***	(0.73– 0.86)
15–16 years old	0.86***	(0.80– 0.93)
17 years old or older	0.83	(0.71– 1.99)
**Male**		
8 years old or younger	0.88	(0.79– 1.07)
9–10 years old	0.73***	(0.65– 0.83)
11–12 years old	0.79***	(0.72– 0.86)
13–14 years old	0.79***	(0.73– 0.85)
15–16 years old	0.83***	(0.77– 0.90)
17 years old or older	0.91	(0.76– 1.07)
**Race/Ethnicity**		
**White** ^b^		
8 years old or younger	0.80***	(0.71– 0.90)
9–10 years old	0.67***	(0.59– 0.77)
11–12 years old	0.78***	(0.71– 0.84)
13–14 years old	0.79***	(0.74– 0.85)
15–16 years old	0.85***	(0.79– 0.91)
17 years old or older	0.83*	(0.70– 0.98)
**Black** ^b^		
8 years old or younger	0.89	(0.69– 1.14)
9–10 years old	0.72*	(0.54– 0.96)
11–12 years old	0.61***	(0.49– 0.76)
13–14 years old	0.68***	(0.57– 0.80)
15–16 years old	0.81*	(0.67– 0.97)
17 years old or older	1.06	(0.68– 1.65)
**Hispanic**		
8 years old or younger	1.05	(0.86– 1.28)
9–10 years old	0.94	(0.78– 1.13)
11–12 years old	0.89	(0.76– 1.03)
13–14 years old	0.90	(0.80– 1.01)
15–16 years old	0.89	(0.76– 1.04)
17 years old or older	0.94	(0.77– 1.16)
**Other Race/Ethnicity**		
8 years old or younger	0.74	(0.59– 1.93)
9–10 years old	0.76*	(0.59– 0.98)
11–12 years old	0.80*	(0.66– 0.95)
13–14 years old	0.87	(0.75– 1.01)
15–16 years old	0.79	(0.66– 1.93)
17 years old or older	0.54	(0.38– 1.79)
**State Dispensary Status**		
**Dispensaries Allowed**		
8 years old or younger	0.90	(0.80– 1.01)
9–10 years old	0.71***	(0.63– 0.80)
11–12 years old	0.74***	(0.68– 0.81)
13–14 years old	0.75***	(0.69– 0.82)
15–16 years old	0.77***	(0.72– 0.84)
17 years old or older	0.82**	(0.71– 0.95)
**Dispensaries Active**		
8 years old or younger	0.93	(0.84– 1.03)
9–10 years old	0.76***	(0.68– 0.85)
11–12 years old	0.84***	(0.77– 0.92)
13–14 years old	0.89**	(0.83– 0.96)
15–16 years old	0.98	(0.92– 1.05)
17 years old or older	1.01	(0.88– 1.16)

* p<.05,

** <.01,

*** p<.001

a State and year variables included but not shown

b Non-Hispanic
